# Visualization of deformation-induced changes in carbon nanotube networks in rubber composites using lock-in thermography†

**DOI:** 10.1039/d3ra00717k

**Published:** 2023-04-17

**Authors:** Naoyuki Matsumoto, Hideaki Nakajima, Takahiro Morimoto, Takeo Yamada, Toshiya Okazaki, Ken Kokubo

**Affiliations:** a Nano Carbon Device Research Center, National Institute of Advanced Industrial Science and Technology (AIST) Tsukuba 305-8565 Japan matsumoto-naoyuki@aist.go.jp

## Abstract

In this study, we used the lock-in thermography technique (LIT) to successfully visualize the single-walled carbon nanotube (CNT) networks during the tensile deformation of CNT/fluoro-rubber (FKM) composites. The LIT images revealed that the CNT network modes in CNT/FKM during strain-loading and unloading can be classified into four sites: (i) disconnection, (ii) recovery after disconnection, (iii) undestroyable, and (iv) no network. Quantitative analysis of the heat intensity of the LIT also indicated that the change in resistance during strain-loading and unloading plays a role in the balance of disconnection and reconstruction of the conductive network. We demonstrated the ability of LIT to effectively visualize and quantify the network state of the composite under deformation, and the LIT results were found to be strongly correlated with the composite properties. These results highlighted the potential of LIT as a valuable tool for composite characterization and material design.

## Abstract 2

In this study, we used the lock-in thermography technique (LIT) to successfully visualize the single-walled carbon nanotube (CNT) networks during the tensile deformation of CNT/fluoro-rubber (FKM) composites.

## Graphical abstract

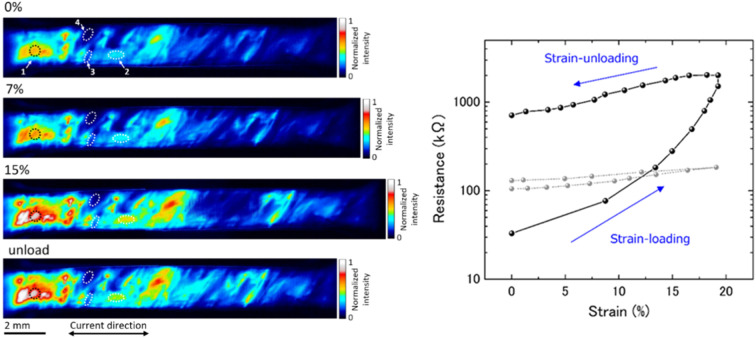

## 1 Introduction

Conductive polymer composites play a crucial role in the development of flexible devices, including applications such as biological sensors, thermal interface material, electromagnetic shielding, and electronic skin.^1–4^ These composites typically achieve their electrical and thermal conductivity by incorporating conductive nanofillers, such as metal fillers (silver and copper), metal oxide fillers (tin oxide and zinc oxide), and carbon fillers (carbon black, carbon fiber, and graphene) into a polymer matrix.^5^ Among these, carbon nanotubes (CNTs) are particularly popular as conductive nanofillers due to their high conductivity, high specific surface area, and high aspect ratio. However, challenges such as achieving proper CNT dispersion within the polymer matrix and maintaining conductivity under deformation continue to limit their use in various applications.

The performance of a flexible device is highly dependent on several critical factors, including sensitivity, durability, and reversibility of resistance to dynamic cycling. These attributes are closely linked to the following two processes. Firstly, as the strain-loading on the polymer material increases, the resistance of the polymer increases non-linearly. This behavior is thought to be due to the disruption or deformation of the network structure created by the conductive fillers within the polymer and has been supported by theoretical analysis.^6,7^ Secondly, as the strain on the polymer material is unloaded, the resistance of the polymer gradually decreases. However, for many conductive polymers with some conductive filler, the resistance after strain-unloading does not return to its original level: the resistance remains higher than it was before the deformation.^6,7^ Although there have been many reported instances of this phenomenon, the specific mechanisms behind it, including the effect of the network structure during strain-loading and unloading, have not yet been fully understood.^6–8^ To develop conductive polymer materials that are highly stable against dynamic cycling, a more detailed analysis of the filler network structure and its relationship to resistance behavior under strain is needed. This can pave the way for the design and synthesis of new conductive polymer materials with improved properties for various applications.

Lock-in thermography (LIT) is a non-destructive method for quickly and accurately assessing the electrical characteristics of large-area samples of bulk materials. By detecting Joule heat during bias application, LIT can quickly image localized electrical defects without being affected by heat storage components. Furthermore, its large field of view, ranging from several millimeters to several centimeters, makes it an ideal tool for evaluating large-scale semiconductors such as solar cells.^9^ Local current density and resistance distribution of local structures at the nanometer scale can also be studied using scanning gate microscopy or conductive atomic force microscopy, which are not well-suited for obtaining high-resolution measurements on large-area samples.^10,11^ Furthermore, various techniques have been reported to visualize CNT fillers in the composites, including impulse acoustic microscopy and infrared thermography non-destructive testing (IR-NDT).^12–15^ However, these methods only allow the evaluation of CNT aggregates and cannot measure individual nanoscale CNTs or bundle-sized networks with high resolution. In addition, these techniques primarily measure the dispersion state on the sample surface, and 3D information about the CNT dispersion state can only be obtained by gradually grinding off the sample by milling or other means. In contrast, LIT allows the observation of CNT networks at the nanoscale with a wide measurement range. This method can measure not only surface (planar) information, but also thickness (3D) information, and can be applied to samples ranging from bulk to microscale. In addition, LIT can simultaneously measure the electrical properties of the sample, allowing the correlation between conductivity and conductive pathways to be visualized and interpreted, which is particularly important during material deformation. Furthermore, LIT can provide not only numerical information but also mapping information that can be used as training data for digital image analysis and machine learning. We have demonstrated that LIT can effectively characterize the CNT network in centimeter-scale CNT/rubber and resin composites.^16,17^ LIT is an excellent evaluation technique for observing the dispersion and conductive behavior of CNT fillers in CNT composites. Its unique ability to visualize conductive networks at the nanoscale, simultaneously measure electrical properties, and provide both numerical and mapping information makes it a powerful tool for studying the dispersion state of CNTs in 3D, including during material deformation.

In this study, we used LIT to conduct time-series imaging measurements of the strain resistance of CNT/rubber composites. Our results show that LIT is capable of visualizing the CNT conductive networks in the CNT/rubber composite under deformation. Additionally, we were able to identify the increased resistance in the strain-loading/unloading process caused by the balance between the disconnection and recovery of the CNT network. These results suggested that LIT is a valuable tool for understanding the underlying mechanisms of strain resistance in CNT/polymer composites and can be used to optimize the design and synthesis of these materials for various applications.

## 2 Experimental

### 2.1 Sample preparation

**Fig. 1:**
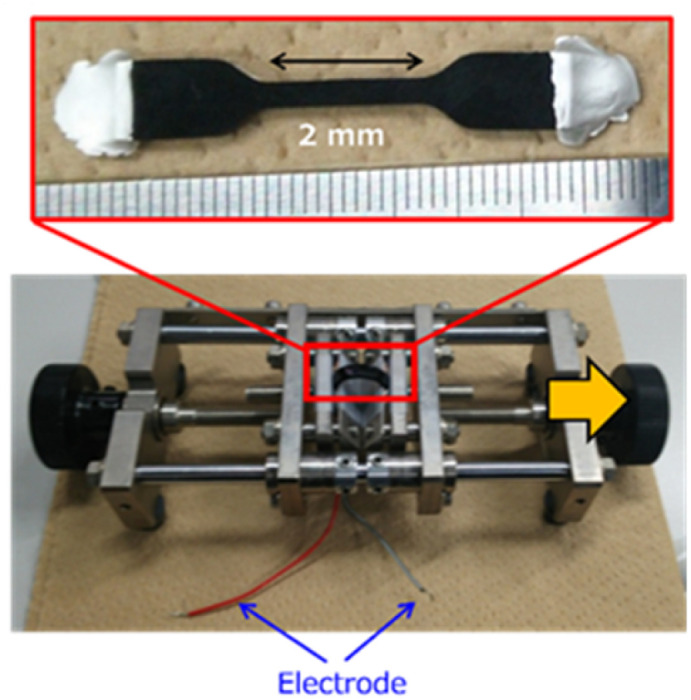
CNT/FKM composite sample and jig used for applying strain (arrows indicate the direction of strain application).

LIT analysis was performed on CNT/rubber composites consisting of super-growth single-walled carbon nanotubes (SWCNTs) and fluoro-rubber (FKM, Daiel G912, Daikin Industries, Ltd). The SWCNTs and CNT/FKM composites were prepared as previously reported.^18,19^ The CNT concentration in the composite was 2.0 wt% of the FKM rubber. The composite was fabricated by compression molding using a mold to produce dumbbell-shaped specimens (35 ± 0.5 mm × 5.0 ± 0.1 mm × 2.0 ± 0.1 mm, strain range: 2.0 ± 0.1 mm) as shown in Fig. 1.

### 2.2 LIT analysis

LIT measurements were performed using a lock-in thermography system for failure analysis (Themos-1000, Hamamatsu). The system's InSb camera detected IR signals with wavelengths from 3.0 to 5.0 μm. The electrical transport characteristics under a DC bias were evaluated using a B1500 semiconductor parameter analyzer (Keysight). Electrodes were made from silver conductive paste, and electrical probing was done using a homemade system. The strain was applied at the desired rate by moving the jig to the right as shown in Fig. 1.

In the strain-unloading process, the strain was gradually returned to the original point (strain = 0%). At each strain point, resistance was measured, and LIT images were observed. The AC bias voltages were applied as square wave signals with a 50% duty cycle. All measurements were made at a frequency of 25 Hz and a peak bias voltage of 40–100 V.

## 3 Results and discussion

### 3.1 Resistance changes of CNT/FKM composites with strain-loading/unloading

**Fig. 2:**
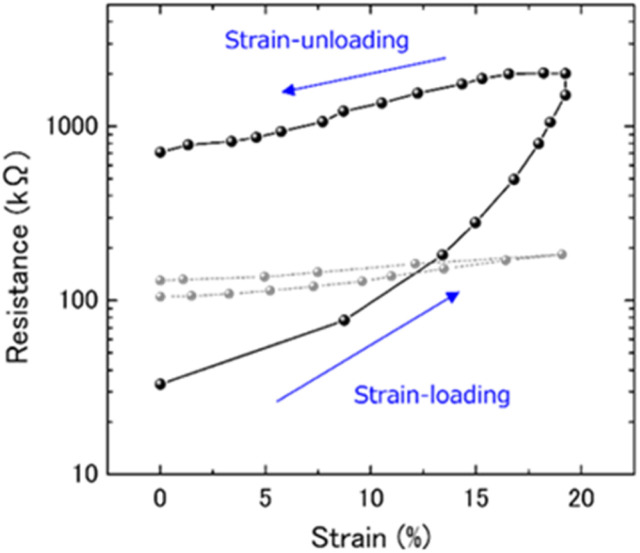
Electrical resistance changes of CNT/FKM composite during strain-loading and unloading. Gray plots show the change in resistivity of uncross-linked CNT/FKM composites.

Fig. 2 shows the change in resistance of CNT/FKM composites with and without cross-linking (grey and black lines respectively) under strain. As the strain increased from 0 to 19%, the resistance of both composites increased, indicating a reduction in the conductivity of the composites. Subsequently, as the strain was gradually released, the resistance also gradually decreased until it reached 0% strain. However, the resistance after strain release was still higher than the initial resistance, whether the composites were crosslinked or not. These results were consistent with those obtained for CNT composites based on natural rubber.^6–8^ In addition, it was observed that the change in resistance was more pronounced in the cross-linked CNT/FKM composites than in the uncross-linked ones.

### 3.2 Visualization of CNT network in CNT/FKM composites

**Fig. 3:**
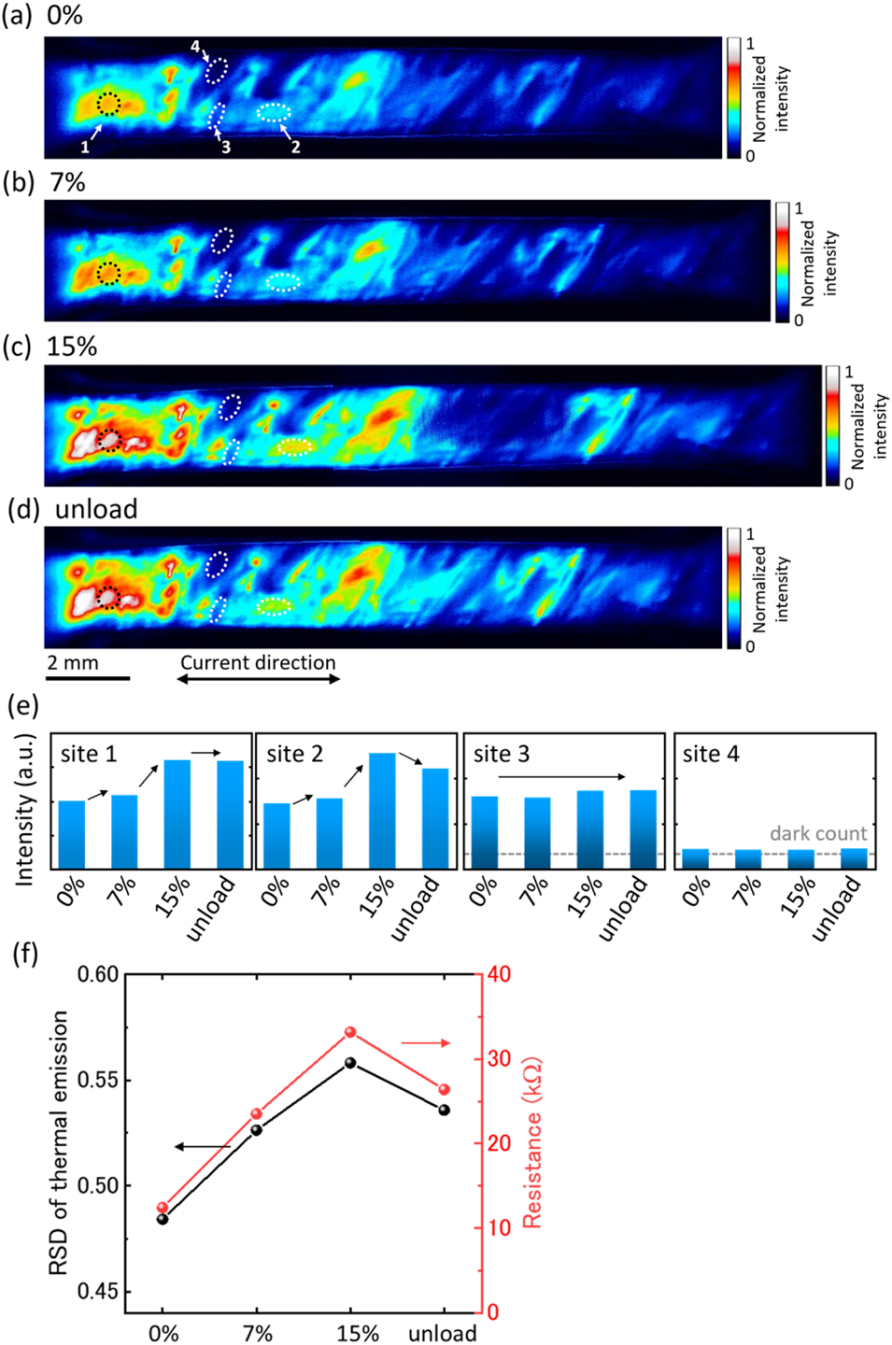
LIT image of CNT/FKM composite at each strain: (a) 0% (no strain), (b) 7.0%, (c) 15%, (d) strain-unloading. (e) Normalization intensity change at each site (sites 1–4) of the LIT image during the strain-loading/unloading process. (f) Relationship between RSD of thermal intensity and electrical resistance of CNT/FKM composite at each strain.

First, the changes in the conductive CNT network in CNT/FKM composites caused by strain-loading and unloading were visualized using LIT. Fig. 3 shows LIT images during strain-loading Fig. 3(a)–(c) and strain-unloading Fig. 3(d). The intensity in Fig. 3 is normalized to the total *W* and is proportional to the power consumption (Joule heat = *I*^2^*R*), which corresponds to the local power concentration. In other words, if the resistive components can be expressed (approximated) by a series connection, and there are no parallel networks of equal or lower resistance, CNT network disconnection is visualized at locations where the heat intensity is high.

Before strain-loading (strain: 0%), a non-uniform heat generation distribution was observed in Fig. 3(a). This result indicates that disconnection of the CNT network is already present during composite fabrication, which affects the initial electrical resistance.

As the strain is applied (Fig. 3(b) and (c)), the heat intensity of the heated area before loading increases, indicating that strain loading (deformation) further leads to the disconnection of the CNT networks. The heat intensity was maintained or decreased in many locations after strain-unloading, as shown in Fig. 3(d). These visualizations of CNT networks using LIT (Fig. 3(a)–(d)) showed that the changes in heat intensity during the strain-loading/unloading process (Fig. 3(e)) can be classified into the following four sites.

• Site 1 (unrecoverable disconnection): the heat intensity increases with strain-loading and remains unchanged upon strain-unloading. This corresponds to the fact that the CNT network is disconnected by strain-loading and remains destroyed even after strain-unloading.

• Site 2 (recovery after disconnection): the heat intensity increases with strain and decreases with strain-unloading, indicating that the CNT networks are initially disconnected during strain-loading, similar to site 1, but are reconstructed upon strain-unloading.

• Site 3 (undestroyable): there is no change in the heat intensity with strain-loading/unloading, indicating that the CNT networks are maintained during strain-loading/unloading. This phenomenon can be attributed to the formation of “flexible” and/or “rigid” CNT network structures, or to the localized presence of high concentrations of CNTs that do not deform the CNT networks during deformation.

• Site 4 (no network): there is no heat intensity (dark areas in the LIT images) during the strain-loading/unloading process. This corresponds to the matrix (rubber) regions where no CNT networks were originally formed during composite fabrication, and thus no Joule heat is generated.

To examine the relationship between the CNT networks and the resistance of the CNT/FKM composite, the relative standard deviation (RSD) of the heating intensity distribution across the sample in the LIT image is plotted in Fig. 3(f). As shown in Fig. 3(f), the change in RSD follows the change in bulk (composite) resistance and is consistent with the results of the LIT image and LIT intensity comparison evaluation shown in Fig. 3(a)–(c). Both the RSD of the heat intensity and the resistance of the CNT/FKM composite increase with an increase in strain, which is attributed to the increased non-uniformity of the heat intensity due to the fracture of the CNT network (site 1). On the other hand, these RSDs and resistances decreased slightly with strain-unloading, which can be attributed to the recovery of the CNT network (site 2). These results suggest that the increase or decrease in resistance with deformation (strain loading/unloading) of the CNT/FKM composite is mainly due to the balance between destruction and reconstruction (sites 1 and 2) of the CNT conductive network.

In Fig. 2, it is observed that the change in resistivity of the CNT/FKM composite before crosslinking is smaller than that after crosslinking. Crosslinking, which is achieved by peroxide vulcanization, increases the mechanical strength of the rubber matrix by forming covalent bonds between the polymer chains, which reduces its flexibility. As a result, the degree of freedom of the CNT filler in the composite is limited by the reduced flexibility of the matrix. This has a significant impact on site 2, as the CNT network is less likely to be reconstructed after crosslinking, resulting in a change in resistance. These results demonstrated that the crosslinking process plays a crucial role in determining the strain resistance of CNT/polymer composites and that by controlling the degree of crosslinking degree, the strain resistance of CNT/polymer composites can be optimized for different applications.

The Digital Image Correlation (DIC) method has been used to observe the deformation (applied strain) of CNT composites.^6^ Although there are examples of strain observation, the behavior of CNT networks has not been directly evaluated by visual observation, but only by the results of strain or conductivity measurements. In contrast, pulse acoustic microscopy and IR-NDT have been used to evaluate CNT aggregates and dispersion in CNT composites, respectively.^12–15^ However, observations of deformation and CNT trajectories with high resolution and information in the 3D direction have not been made. Therefore, a direct relationship between CNT network structure and conductivity during deformation has not been established.

In this study, we aimed to evaluate the first application of strain where the change (increase) in resistance is greatest, and where the CNT network is most susceptible to disconnection. The increase in resistance (decrease in electrical conductivity) with applied strain in CNT composites has previously been attributed to CNT network disconnection throughout the sample.^6^ However, by using LIT to visualize the CNT network of the entire composite at high resolution, we found that CNT disconnection occurs in a part of the composite and can be further categorized by the degree of connectivity of the CNT network (Fig. 3(a)–(d)). We have shown for the first time that the CNT network is not uniformly disconnected throughout the composite, as the poor connection of the CNT network is further weakened by the application of strain.

Quantitative analysis of statistically processed LIT images (including image processing) shows strong correlations with composite properties, indicating that LIT is an evaluation tool that links filler network structure to physical properties with or without composite deformation. However, a combined evaluation using SEM, TEM, and AFM, such as how the CNT network is formed, is required for areas of disconnection, recovery, and undestroyable CNT networks revealed by LIT, which is possible because LIT is non-destructive. Furthermore, it would be possible to increase the optimal CNT dispersion state (site 3) based on the correlation between the process (mixing and molding conditions) and the conductive network by LIT.

## 4 Conclusions

We employed the LIT to non-destructively evaluate the changes in the CNT network in CNT/FKM rubber composites under deformation. We have clarified the mechanism by which deformation causes the CNT network to fracture and restructure, and the balance of these changes the electrical resistance of the composite. Based on the revealed mechanisms, it is important to select materials and processes that improve CNT contact defects and produce CNT network structures with uniform strength or flexibility against deformation to produce CNT/FKM composites that retain their properties during deformation. LIT is a powerful evaluation method for understanding conductive filler networks in composites under deformation and has potential applications in the development of advanced human sensors and other devices that repeatedly undergo complex deformations due to human motion.

## Author contributions

N. M., H. N., T. M., and T. O. designed the experiments. N. M. prepared the CNT/FKM composites. H. N. measured the resistivity and observed the LIT images. N. M. and H. N. analyzed all measurement data and then wrote the manuscript. All authors discussed the results and revised the manuscript.

## Conflicts of interest

The authors declare that we have no competing financial interests or personal relationships that could have appeared to influence the work reported in this paper.

## Supplementary Material

RA-013-D3RA00717K-s001
