# Supplementary figures and images for: Visualization of deformation-induced changes in carbon nanotube networks in rubber composites using lock-in thermography
Source: RSC Adv. 2023 Apr 17;13(18):11884–8. doi: 10.1039/d3ra00717k (PMC10108577; doi:10.1039/d3ra00717k)

## Supplemental Figure

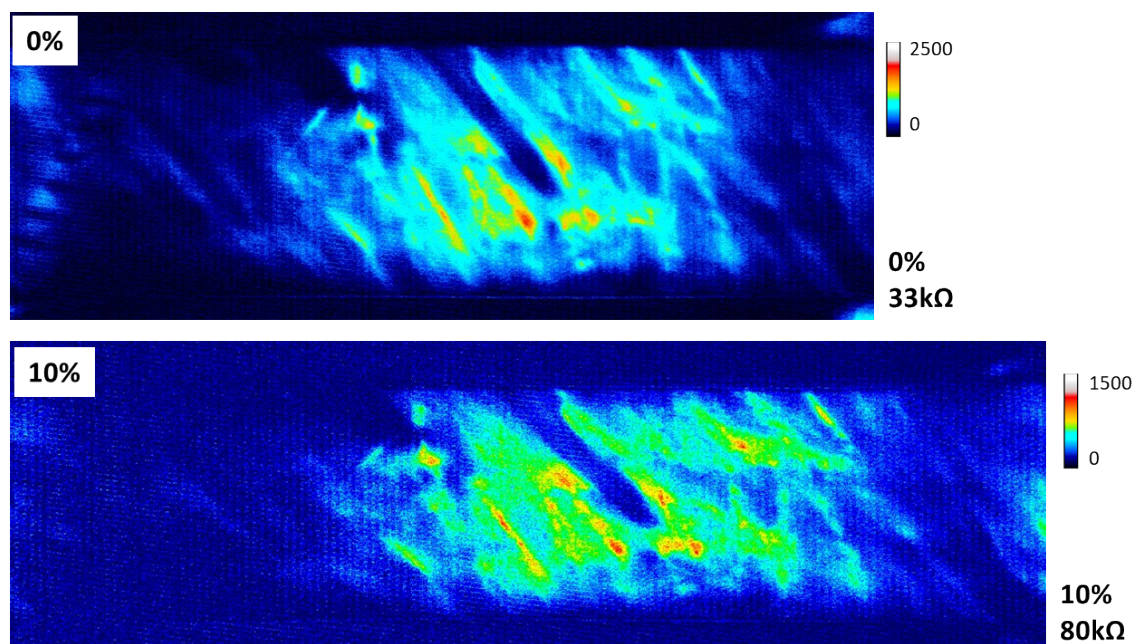

Fig. S1 LIT images of another CNT/FKM composite.

Supplement: RA-013-D3RA00717K-s001 [file RA-013-D3RA00717K-s001.pdf]
